# Diethyl 2,6-dimethyl-4-(4-pyrid­yl)-1,4-dihydro­pyridine-3,5-dicarboxyl­ate

**DOI:** 10.1107/S1600536810016934

**Published:** 2010-05-15

**Authors:** Yumei Li

**Affiliations:** a Department of Chemistry, Dezhou University, Shandong 253023, People’s Republic of China

## Abstract

In the title compound, C_18_H_22_N_2_O_4_, the dihedral angle between the two rings is 87.90 (6)°. The mean devation of the atoms in the dihydropyridine plane is 0.082 (3) Å. In the crystal, mol­ecules are linked by inter­molecular N—H⋯N hydrogen bonds, generating chains.

## Related literature

For general background to the biological activity of 1,4-dihydro­pyridine derivatives, see: Gaudio *et al.* (1994 ▶).
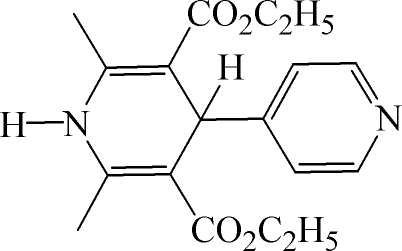


## Experimental

### 

#### Crystal data

C_18_H_22_N_2_O_4_
*M* *_r_* = 330.38
Monoclinic, 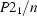

*a* = 11.5550 (2) Å
*b* = 13.1707 (2) Å
*c* = 11.8020 (2) Å
β = 92.705 (2)°
*V* = 1794.11 (5) Å^3^
*Z* = 4
Mo *K*α radiation
μ = 0.09 mm^−1^
*T* = 296 K
0.12 × 0.10 × 0.08 mm

#### Data collection

Bruker APEXII CCD diffractometer
Absorption correction: multi-scan (*SADABS*; Bruker, 2001 ▶) *T*_min_ = 0.990, *T*_max_ = 0.993
9122 measured reflections
3152 independent reflections
2308 reflections with *I* > 2σ(*I*)
*R*_int_ = 0.032

#### Refinement

*R*[*F* ^2^ > 2σ(*F* ^2^)] = 0.045
*wR*(*F* ^2^) = 0.128
*S* = 1.03
3152 reflections
227 parameters
H atoms treated by a mixture of independent and constrained refinement
Δρ_max_ = 0.15 e Å^−3^
Δρ_min_ = −0.19 e Å^−3^

### 

Data collection: *APEX2* (Bruker, 2004 ▶); cell refinement: *SAINT-Plus* (Bruker, 2001 ▶); data reduction: *SAINT-Plus*; program(s) used to solve structure: *SHELXS97* (Sheldrick, 2008 ▶); program(s) used to refine structure: *SHELXL97* (Sheldrick, 2008 ▶); molecular graphics: *SHELXTL* (Sheldrick, 2008 ▶); software used to prepare material for publication: *SHELXL97*.

## Supplementary Material

Crystal structure: contains datablocks global, I. DOI: 10.1107/S1600536810016934/om2336sup1.cif

Structure factors: contains datablocks I. DOI: 10.1107/S1600536810016934/om2336Isup2.hkl

Additional supplementary materials:  crystallographic information; 3D view; checkCIF report

## Figures and Tables

**Table 1:** Hydrogen-bond geometry (Å, °)

*D*—H⋯*A*	*D*—H	H⋯*A*	*D*⋯*A*	*D*—H⋯*A*
N2—H2*N*⋯N1^i^	0.86 (2)	2.13 (2)	2.984 (2)	171.8 (18)

Symmetry code: (i) 
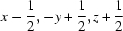
.

## supplementary crystallographic information

### Comment

The synthesis of 1,4-dihydropyridine derivatives has attracted continuous research interest due to various vasodilator, anti-hypertensive, bronchodilator, heptaprotective, anti-tumor, anti-mutagenic, geroprotective and anti-diabetic agents (Gaudio *et al.*, 1994).

The molecular structure of the title compound is shown in Fig 1. The dihedral angle between the two rings is 87.90 (6) °. The mean devation of the dihydropyridine plane is 0.082 (3)Å. The intermolecular hydrogen bonding of N(2)—H(2A)···N(1) leads to a consolidation of the structure (Fig. 2; Table 1).

### Experimental

Diethyl 2,6-dimethyl-4-(4-pyridyl)-1,4-dihydropyridine-3,5-dicarboxylate was purchased from Jinan Henghua Science & Technology Co. Ltd. Diethyl 2,6-dimethyl-4-(4-pyridyl)-1,4-dihydropyridine-3,5-dicarboxylate (1 mmoL 0.39 g) was dissolved in 20 ml ethanol, which was evaporated in an open flask at room temperature. One week later, yellow block crystals suitable for the X-ray experiment were obained. Anal. C_18_H_22_N_2_O_4_: C, 65.37; H, 6.66; N, 8.48 %. Found: C, 65.32; H, 6.45; N, 8.39 %.

### Refinement

All hydrogen atoms bound to aromatic carbon atoms were refined in calculated positions using a riding model with a C—H distance of 0.93 Å and *U*_iso_ = 1.2*U*_eq_(C). For methyl groups C—H distances were 0.96 Å and *U*_iso_ = 1.5*U*_eq_(C) . Two of the methyl groups were found to have two sets of methyl hydrogens and were refined with AFIX 127 and major part occupanices that refined to 0.60 (2) and 0.59 (2) for C17 and C18, respectively.  The hydrogen atom attached to the hydropyridine nitrogen was freely refined.

### Crystal data

**Table:**

C_18_H_22_N_2_O_4_	*F*(000) = 704
*M**_r_* = 330.38	*D*_x_ = 1.223 Mg m^−^^3^
Monoclinic, *P*2_1_/*n*	Mo *K*α radiation, λ = 0.71073 Å
Hall symbol: -P 2yn	Cell parameters from 2361 reflections
*a* = 11.5550 (2) Å	θ = 2.3–24.5°
*b* = 13.1707 (2) Å	µ = 0.09 mm^−^^1^
*c* = 11.8020 (2) Å	*T* = 296 K
β = 92.705 (2)°	Block, yellow
*V* = 1794.11 (5) Å^3^	0.12 × 0.10 × 0.08 mm
*Z* = 4	

### Data collection

**Table:**

Bruker APEXII CCD diffractometer	3152 independent reflections
Radiation source: fine-focus sealed tube	2308 reflections with *I* > 2σ(*I*)
graphite	*R*_int_ = 0.032
φ and ω scans	θ_max_ = 25.0°, θ_min_ = 2.3°
Absorption correction: multi-scan (*SADABS*; Bruker, 2001)	*h* = −11→13
*T*_min_ = 0.990, *T*_max_ = 0.993	*k* = −15→15
9122 measured reflections	*l* = −14→13

### Refinement

**Table:**

Refinement on *F*^2^	Primary atom site location: structure-invariant direct methods
Least-squares matrix: full	Secondary atom site location: difference Fourier map
*R*[*F*^2^ > 2σ(*F*^2^)] = 0.045	Hydrogen site location: inferred from neighbouring sites
*wR*(*F*^2^) = 0.128	H atoms treated by a mixture of independent and constrained refinement
*S* = 1.03	*w* = 1/[σ^2^(*F*_o_^2^) + (0.0645*P*)^2^ + 0.2873*P*] where *P* = (*F*_o_^2^ + 2*F*_c_^2^)/3
3152 reflections	(Δ/σ)_max_ = 0.001
227 parameters	Δρ_max_ = 0.15 e Å^−^^3^
0 restraints	Δρ_min_ = −0.19 e Å^−^^3^

### Special details

**Table:**

Geometry. All esds (except the esd in the dihedral angle between two l.s. planes) are estimated using the full covariance matrix. The cell esds are taken into account individually in the estimation of esds in distances, angles and torsion angles; correlations between esds in cell parameters are only used when they are defined by crystal symmetry. An approximate (isotropic) treatment of cell esds is used for estimating esds involving l.s. planes.
Refinement. Refinement of *F*^2^ against ALL reflections. The weighted *R*-factor wR and goodness of fit *S* are based on *F*^2^, conventional *R*-factors *R* are based on *F*, with *F* set to zero for negative *F*^2^. The threshold expression of *F*^2^ > σ(*F*^2^) is used only for calculating *R*-factors(gt) etc. and is not relevant to the choice of reflections for refinement. *R*-factors based on *F*^2^ are statistically about twice as large as those based on *F*, and *R*- factors based on ALL data will be even larger.

### Fractional atomic coordinates and isotropic or equivalent isotropic displacement parameters (Å^2^)

**Table:**

	*x*	*y*	*z*	*U*_iso_*/*U*_eq_	Occ. (<1)
O1	0.33862 (13)	0.01201 (15)	0.15684 (14)	0.0917 (6)	
O2	0.50534 (12)	−0.07176 (10)	0.16753 (11)	0.0597 (4)	
O3	0.80150 (10)	0.08444 (10)	0.58574 (10)	0.0539 (4)	
O4	0.81559 (11)	−0.01782 (11)	0.43654 (12)	0.0630 (4)	
N1	0.82662 (14)	0.18151 (15)	0.07048 (14)	0.0616 (5)	
N2	0.47955 (13)	0.18372 (12)	0.43985 (13)	0.0472 (4)	
H2N	0.4391 (17)	0.2279 (15)	0.4751 (16)	0.058 (6)*	
C1	0.76016 (19)	0.23648 (17)	0.13643 (17)	0.0626 (6)	
H1	0.7595	0.3066	0.1272	0.075*	
C2	0.69267 (17)	0.19512 (14)	0.21726 (16)	0.0527 (5)	
H2	0.6482	0.2374	0.2610	0.063*	
C3	0.69017 (13)	0.09216 (12)	0.23420 (13)	0.0376 (4)	
C4	0.75744 (16)	0.03451 (15)	0.16496 (15)	0.0525 (5)	
H4	0.7586	−0.0358	0.1717	0.063*	
C5	0.82298 (17)	0.08201 (18)	0.08560 (17)	0.0631 (6)	
H5	0.8674	0.0415	0.0399	0.076*	
C6	0.76250 (15)	0.04661 (14)	0.48574 (14)	0.0431 (4)	
C7	0.91311 (16)	0.04589 (17)	0.62964 (17)	0.0601 (5)	
H7A	0.9739	0.0664	0.5806	0.072*	
H7B	0.9116	−0.0277	0.6328	0.072*	
C8	0.9350 (2)	0.0879 (2)	0.7438 (2)	0.0949 (9)	
H8A	0.9386	0.1606	0.7394	0.142*	
H8B	1.0072	0.0622	0.7754	0.142*	
H8C	0.8734	0.0684	0.7911	0.142*	
C9	0.43413 (16)	0.00167 (16)	0.20127 (15)	0.0514 (5)	
C10	0.4620 (2)	−0.13703 (19)	0.0762 (2)	0.0795 (7)	
H10A	0.4463	−0.0974	0.0079	0.095*	
H10B	0.3905	−0.1694	0.0967	0.095*	
C11	0.5496 (3)	−0.2133 (3)	0.0566 (3)	0.1604 (18)	
H11A	0.6205	−0.1805	0.0384	0.241*	
H11B	0.5236	−0.2562	−0.0053	0.241*	
H11C	0.5624	−0.2536	0.1238	0.241*	
C12	0.61631 (13)	0.04467 (13)	0.32414 (13)	0.0373 (4)	
H12	0.6308	−0.0286	0.3252	0.045*	
C13	0.48786 (14)	0.06167 (13)	0.29414 (14)	0.0405 (4)	
C14	0.42771 (14)	0.13185 (13)	0.34979 (14)	0.0424 (4)	
C15	0.58549 (14)	0.15725 (13)	0.49037 (14)	0.0425 (4)	
C16	0.65116 (13)	0.08686 (12)	0.44024 (13)	0.0381 (4)	
C17	0.61248 (18)	0.21403 (17)	0.59884 (16)	0.0614 (6)	
H17A	0.6361	0.1669	0.6575	0.092*	0.60 (2)
H17B	0.5447	0.2499	0.6205	0.092*	0.60 (2)
H17C	0.6739	0.2615	0.5878	0.092*	0.60 (2)
H17D	0.6004	0.2854	0.5864	0.092*	0.40 (2)
H17E	0.6918	0.2023	0.6233	0.092*	0.40 (2)
H17F	0.5625	0.1907	0.6561	0.092*	0.40 (2)
C18	0.30334 (15)	0.16157 (17)	0.32620 (17)	0.0584 (5)	
H18A	0.2596	0.1466	0.3913	0.088*	0.59 (2)
H18B	0.2721	0.1242	0.2620	0.088*	0.59 (2)
H18C	0.2989	0.2330	0.3102	0.088*	0.59 (2)
H18D	0.2941	0.1892	0.2511	0.088*	0.41 (2)
H18E	0.2816	0.2116	0.3804	0.088*	0.41 (2)
H18F	0.2548	0.1028	0.3321	0.088*	0.41 (2)

### Atomic displacement parameters (Å^2^)

**Table:**

	*U*^11^	*U*^22^	*U*^33^	*U*^12^	*U*^13^	*U*^23^
O1	0.0504 (9)	0.1327 (15)	0.0895 (11)	0.0078 (9)	−0.0249 (9)	−0.0411 (11)
O2	0.0560 (8)	0.0669 (9)	0.0552 (8)	−0.0058 (7)	−0.0078 (7)	−0.0177 (7)
O3	0.0388 (7)	0.0738 (9)	0.0480 (7)	0.0130 (6)	−0.0106 (6)	−0.0075 (6)
O4	0.0505 (8)	0.0732 (9)	0.0643 (9)	0.0217 (7)	−0.0080 (7)	−0.0164 (7)
N1	0.0482 (10)	0.0823 (13)	0.0547 (10)	−0.0138 (9)	0.0065 (8)	0.0115 (9)
N2	0.0358 (8)	0.0622 (10)	0.0436 (9)	0.0121 (7)	−0.0003 (7)	−0.0067 (8)
C1	0.0689 (14)	0.0555 (12)	0.0637 (13)	−0.0175 (11)	0.0050 (11)	0.0075 (10)
C2	0.0552 (12)	0.0487 (11)	0.0551 (11)	−0.0048 (9)	0.0113 (9)	−0.0026 (9)
C3	0.0289 (8)	0.0479 (10)	0.0355 (9)	−0.0015 (7)	−0.0038 (7)	−0.0004 (7)
C4	0.0520 (11)	0.0542 (12)	0.0522 (11)	0.0069 (9)	0.0121 (9)	0.0029 (9)
C5	0.0491 (12)	0.0853 (17)	0.0563 (13)	0.0091 (11)	0.0171 (10)	0.0050 (11)
C6	0.0383 (10)	0.0507 (10)	0.0401 (10)	0.0015 (8)	0.0004 (8)	0.0003 (8)
C7	0.0367 (10)	0.0759 (14)	0.0662 (13)	0.0114 (10)	−0.0129 (9)	−0.0020 (11)
C8	0.0617 (15)	0.144 (2)	0.0762 (16)	0.0252 (16)	−0.0278 (13)	−0.0237 (17)
C9	0.0433 (11)	0.0666 (13)	0.0441 (11)	−0.0083 (9)	0.0010 (9)	−0.0025 (9)
C10	0.0852 (17)	0.0833 (17)	0.0684 (14)	−0.0116 (14)	−0.0149 (13)	−0.0286 (13)
C11	0.172 (4)	0.139 (3)	0.164 (3)	0.065 (3)	−0.066 (3)	−0.101 (3)
C12	0.0350 (9)	0.0402 (9)	0.0366 (9)	−0.0001 (7)	0.0009 (7)	0.0014 (7)
C13	0.0342 (9)	0.0510 (10)	0.0362 (9)	−0.0058 (8)	0.0011 (7)	0.0040 (8)
C14	0.0329 (9)	0.0555 (11)	0.0386 (9)	−0.0012 (8)	0.0011 (7)	0.0066 (8)
C15	0.0363 (9)	0.0543 (11)	0.0366 (9)	0.0025 (8)	−0.0002 (8)	0.0015 (8)
C16	0.0324 (9)	0.0443 (10)	0.0374 (9)	0.0013 (7)	−0.0002 (7)	0.0008 (7)
C17	0.0537 (12)	0.0806 (14)	0.0493 (11)	0.0159 (10)	−0.0048 (9)	−0.0170 (10)
C18	0.0339 (10)	0.0825 (14)	0.0586 (12)	0.0053 (9)	−0.0019 (9)	0.0038 (11)

### Geometric parameters (Å, °)

**Table:**

O1—C9	1.207 (2)	C9—C13	1.465 (2)
O2—C9	1.342 (2)	C10—C11	1.452 (4)
O2—C10	1.449 (2)	C10—H10A	0.9700
O3—C6	1.339 (2)	C10—H10B	0.9700
O3—C7	1.458 (2)	C11—H11A	0.9600
O4—C6	1.211 (2)	C11—H11B	0.9600
N1—C5	1.324 (3)	C11—H11C	0.9600
N1—C1	1.333 (3)	C12—C16	1.515 (2)
N2—C14	1.377 (2)	C12—C13	1.526 (2)
N2—C15	1.381 (2)	C12—H12	0.9800
N2—H2N	0.86 (2)	C13—C14	1.346 (2)
C1—C2	1.373 (3)	C14—C18	1.503 (2)
C1—H1	0.9300	C15—C16	1.352 (2)
C2—C3	1.371 (2)	C15—C17	1.503 (2)
C2—H2	0.9300	C17—H17A	0.9600
C3—C4	1.381 (2)	C17—H17B	0.9600
C3—C12	1.527 (2)	C17—H17C	0.9600
C4—C5	1.382 (3)	C17—H17D	0.9600
C4—H4	0.9300	C17—H17E	0.9600
C5—H5	0.9300	C17—H17F	0.9600
C6—C16	1.470 (2)	C18—H18A	0.9600
C7—C8	1.467 (3)	C18—H18B	0.9600
C7—H7A	0.9700	C18—H18C	0.9600
C7—H7B	0.9700	C18—H18D	0.9600
C8—H8A	0.9600	C18—H18E	0.9600
C8—H8B	0.9600	C18—H18F	0.9600
C8—H8C	0.9600		
			
C9—O2—C10	116.92 (16)	H11A—C11—H11B	109.5
C6—O3—C7	116.02 (14)	C10—C11—H11C	109.5
C5—N1—C1	115.87 (17)	H11A—C11—H11C	109.5
C14—N2—C15	123.55 (16)	H11B—C11—H11C	109.5
C14—N2—H2N	118.7 (13)	C16—C12—C13	111.71 (13)
C15—N2—H2N	116.8 (13)	C16—C12—C3	110.18 (13)
N1—C1—C2	123.51 (19)	C13—C12—C3	110.37 (13)
N1—C1—H1	118.2	C16—C12—H12	108.2
C2—C1—H1	118.2	C13—C12—H12	108.2
C3—C2—C1	120.68 (18)	C3—C12—H12	108.2
C3—C2—H2	119.7	C14—C13—C9	121.67 (16)
C1—C2—H2	119.7	C14—C13—C12	120.43 (15)
C2—C3—C4	116.16 (16)	C9—C13—C12	117.87 (15)
C2—C3—C12	121.52 (15)	C13—C14—N2	120.11 (15)
C4—C3—C12	122.32 (15)	C13—C14—C18	126.85 (16)
C3—C4—C5	119.59 (18)	N2—C14—C18	113.03 (16)
C3—C4—H4	120.2	C16—C15—N2	119.26 (15)
C5—C4—H4	120.2	C16—C15—C17	128.02 (15)
N1—C5—C4	124.17 (19)	N2—C15—C17	112.72 (15)
N1—C5—H5	117.9	C15—C16—C6	125.97 (15)
C4—C5—H5	117.9	C15—C16—C12	121.10 (14)
O4—C6—O3	121.74 (15)	C6—C16—C12	112.88 (14)
O4—C6—C16	122.14 (16)	C15—C17—H17A	109.5
O3—C6—C16	116.11 (15)	C15—C17—H17B	109.5
O3—C7—C8	107.81 (17)	H17A—C17—H17B	109.5
O3—C7—H7A	110.1	C15—C17—H17C	109.5
C8—C7—H7A	110.1	H17A—C17—H17C	109.5
O3—C7—H7B	110.1	H17B—C17—H17C	109.5
C8—C7—H7B	110.1	C15—C17—H17D	109.5
H7A—C7—H7B	108.5	C15—C17—H17E	109.5
C7—C8—H8A	109.5	H17D—C17—H17E	109.5
C7—C8—H8B	109.5	C15—C17—H17F	109.5
H8A—C8—H8B	109.5	H17D—C17—H17F	109.5
C7—C8—H8C	109.5	H17E—C17—H17F	109.5
H8A—C8—H8C	109.5	C14—C18—H18A	109.5
H8B—C8—H8C	109.5	C14—C18—H18B	109.5
O1—C9—O2	120.87 (17)	H18A—C18—H18B	109.5
O1—C9—C13	127.63 (19)	C14—C18—H18C	109.5
O2—C9—C13	111.50 (15)	H18A—C18—H18C	109.5
O2—C10—C11	108.0 (2)	H18B—C18—H18C	109.5
O2—C10—H10A	110.1	C14—C18—H18D	109.5
C11—C10—H10A	110.1	C14—C18—H18E	109.5
O2—C10—H10B	110.1	H18D—C18—H18E	109.5
C11—C10—H10B	110.1	C14—C18—H18F	109.5
H10A—C10—H10B	108.4	H18D—C18—H18F	109.5
C10—C11—H11A	109.5	H18E—C18—H18F	109.5
C10—C11—H11B	109.5		

### Hydrogen-bond geometry (Å, °)

**Table:**

*D*—H···*A*	*D*—H	H···*A*	*D*···*A*	*D*—H···*A*
N2—H2N···N1^i^	0.86 (2)	2.13 (2)	2.984 (2)	171.8 (18)

Symmetry codes: (i) *x*−1/2, −*y*+1/2, *z*+1/2.
